# BioBenchmark Toyama 2012: an evaluation of the performance of triple stores on biological data

**DOI:** 10.1186/2041-1480-5-32

**Published:** 2014-07-10

**Authors:** Hongyan Wu, Toyofumi Fujiwara, Yasunori Yamamoto, Jerven Bolleman, Atsuko Yamaguchi

**Affiliations:** 1 Database Center for Life Science, Research Organization of Information and Systems, 178-4-4 Wakashiba, Kashiwa, Chiba 277-0871, Japan; 2 INTEC Inc, 1-3-3 Shinsuna, Koto-ku, Tokyo 136-8637, Japan; 3 Swiss-Prot group, SIB Swiss Institute of Bioinformatics, CMU, 1 Michel Servet, 1211 Geneva 4, Switzerland

## Abstract

**Background:**

Biological databases vary enormously in size and data complexity, from small databases that contain a few million Resource Description Framework (RDF) triples to large databases that contain billions of triples. In this paper, we evaluate whether RDF native stores can be used to meet the needs of a biological database provider. Prior evaluations have used synthetic data with a limited database size. For example, the largest BSBM benchmark uses 1 billion synthetic e-commerce knowledge RDF triples on a single node. However, real world biological data differs from the simple synthetic data much. It is difficult to determine whether the synthetic e-commerce data is efficient enough to represent biological databases. Therefore, for this evaluation, we used five real data sets from biological databases.

**Results:**

We evaluated five triple stores, 4store, Bigdata, Mulgara, Virtuoso, and OWLIM-SE, with five biological data sets, Cell Cycle Ontology, Allie, PDBj, UniProt, and DDBJ, ranging in size from approximately 10 million to 8 billion triples.

For each database, we loaded all the data into our single node and prepared the database for use in a classical data warehouse scenario. Then, we ran a series of SPARQL queries against each endpoint and recorded the execution time and the accuracy of the query response.

**Conclusions:**

Our paper shows that with appropriate configuration Virtuoso and OWLIM-SE can satisfy the basic requirements to load and query biological data less than 8 billion or so on a single node, for the simultaneous access of 64 clients.

OWLIM-SE performs best for databases with approximately 11 million triples; For data sets that contain 94 million and 590 million triples, OWLIM-SE and Virtuoso perform best. They do not show overwhelming advantage over each other; For data over 4 billion Virtuoso works best.

4store performs well on small data sets with limited features when the number of triples is less than 100 million, and our test shows its scalability is poor; Bigdata demonstrates average performance and is a good open source triple store for middle-sized (500 million or so) data set; Mulgara shows a little of fragility.

## Background

Semantic Web encodes information from the World Wide Web in a machine-readable syntax to make web information automatically recognizable and processable by computers [1]. Semantic Web, which “is about common formats for integration and combination of data drawn from diverse sources” [2], facilitates the integration of heterogeneous data on the World Wide Web by applying formal ontologies to specify the semantics of the data explicitly [3]. Semantic Web has unleashed a revolution of data publication and interconnection [4].

Semantic Web has gained significance in the life sciences. Due to the success of the Human Genome Project (HGP) [5] and high-throughput sequencing, a large quantity of biological data is available to the scientific community via the Internet [4]. One challenge posed by biological databases is the diversity of data types, which include sequence (e.g., NCBI’s GenBank [6]), microarray gene expression (e.g., SMD [7] and GEO [8]), pathway (e.g., BIND [9]), and proteomic data (e.g., PeptideAtlas [10]). These diverse data types are highly heterogeneous both in structure and semantics [11]. However, the complexity of a disease cannot be explained without referring to multiple biological databases. For example, to understand Parkinson’s disease requires both neuroscience information as well as mapping of gene expression across the whole brain [12,13]. Semantic Web provides a way to integrate heterogeneous data source.

Life and health science communities [14] have made remarkable progress as early adopters of Semantic Web technologies [15]. For example, the UniProt knowledgebase [16] is one of the core public databases in the life sciences. UniProt connects more than 150 molecular biology and chemoinformatics databases and integrates, interprets, and standardizes data from numerous resources to achieve the most comprehensive catalogue of protein sequences and functional annotations. As another example, the Protein Data Bank Japan(PDBj) [17] accepts and processes PDB entries that are deposited mainly from Asian and Oceanic researchers and maintains a centralized archive of macromolecular structures in collaboration with other wwPDB [18] members, including the RCSB-PDB [19], the BMRB [20] in the US, and the PDBe [21] in Europe.

The popularity of Semantic Web has accelerated the rapid development of one of its core techniques, the triple store. A triple store [22] is designed to store and retrieve triples, which is a statement relating one object to another. This paper evaluates the performance of five native triple stores on biological data.

Our evaluation was motivated by a project that is supported by the Japan Science and Technology Agency to integrate data in the life sciences. Our aim is to evaluate whether RDF native stores can meet the needs of a biological database provider. Existing benchmarks, such as the Lehigh University Benchmark (LUBM [23]) and the Berlin SPARQL Benchmark (BSBM [24]), use a data generator to produce synthetic e-commerce knowledge data, and the largest database on a single machine generated by such a data generator includes 1 billion triples. However, real world biological data differs from the simple synthetic data much. The UniProt data has 164 owl classes and uses more than 180 properties, while SP ^2^Bench [25] uses only 23 properties, and BSBM [24] uses a similar number of properties and only 8 classes. Due to one RDF triple including only one property, 180 properties may theoretically need the times of join over 180. This means that both the graph and queries in UniProt are significantly different in form to the generated data in either SP ^2^Bench or BSBM. In addition each instance in RDF may differ much from each other even in the same class, which makes RDF flexible to express heterogeneous data, and therefore to pick up a set of instances covering all 180 properties and 164 classes will take a lot of effort. It is quite difficult to guarantee the conclusions drawn from synthetic benchmarks or other fields are applicable to biological data. The biological data benchmark, Cell Cycle Ontology [26] uses real biological data. However, it includes only 10 million triples. We used five groups of real biological data set ranging from 10 million to 8 billion to make sure that the data was scalable and variable enough. Due to hardware requirement of running a datastore of the UniProt and DDBJ size there are few if any dependable public benchmark results i.e. fully describing the disk system and software used. There are no reports for single node installations with dataset sizes of more than 1 billion nodes. Our target is to verify applicability of a triple store for biological databases.

For this evaluation, we used biological databases, Cell Cycle Ontology, Allie, PDBj, UniProt, and DDBJ containing as many as 8 billion triples. Biological databases are also characterized by diverse and sparse data, which may impact performance. We evaluated the load and query costs of five popular triple stores: 4store, Bigdata, Mulgara, Virtuoso, and OWLIM-SE. To the best of our knowledge, we evaluated the largest scale of real biological data possible on a single node.

## Methods

### Triple store

We selected five native triple stores. Three of them were recommended by the Bioinformaticians in the international symposium Biohackathon 2011, who had used or tested these triple stores for their biological data. 4store was used in the Cell Cycle Ontology [26]. Mulgara was used as an internal triple store in DDBj. OWLIM-SE has been applied as UniProt triple store. Virtuoso showed good performance in BSBM and DBpedia SPARQL Benchmark. Bigdata, a complete free open source triple, performed averagely well in BSBM and supported most of inference functions and could run in both single node and cluster mode. It could be a potentially good candidate to customize one’s own triple store. Neither Jena TDB nor Jena SDB showed attractive performance in [26], in which both of them worked worse than 4store and Virtuoso. Sesame showed bad load performance in BSBM Version 1. We evaluated the triple stores using their newest versions as of June 30, 2012.

#### 4store

4store [27,28] is a RDF/SPARQL store that is written in C and designed to run on UNIX-based systems. 4store can be run on a single machine or networked clusters. We evaluated 4store version 1.1.4.

#### Bigdata

Bigdata [29] is designed as a distributed database architecture that runs on clusters of hundreds to thousands of commodity machines. However, Bigdata can also run in high-performance single-server mode. Bigdata supports RDFS and limited OWL inference. Bigdata is open-source software that is written in Java. We evaluated version RWSTORE_1_1_0.

#### OWLIM-SE

OWLIM-SE [30,31] is a member of the OWLIM family (OWLIM-Lite, OWLIM-SE, OWLIM-Enterprise, and OWLIM on Amazon AWS), which provides native RDF engines that are implemented in Java and deliver full performance through both Sesame and Jena. Beginning with version 4.3, OWLIM-SE supports SPARQL 1.1 Federation. OWLIM-SE also supports the semantics of RDFS, OWL 2 RL, and OWL 2 QL. OWLIM-SE is available by commercial license only. We evaluated OWLIM-SE version 5.1.5269.

#### Mulgara

Mulgara [32] is an open-source triple store that is written in Java. Mulgara provides a SQL-like language shell, iTQL (Interactive Tucana Query Language), to query and update Mulgara databases. Mulgara supports RDFS and OWL inference. In addition, Mulgara also provides a SPARQL query parser and query engine. We evaluated Mulgara version V2.1.13.

#### Virtuoso

Virtuoso [33,34] provides a triple storage solution for RDF on RDBMS platforms. Virtuoso is a multi-purpose data server that supports RDBMS, RDF, and XML. Virtuoso offers stored procedures to load RDFXML, ntriples, and compressed triples. Virtuoso also supports SPARQL as well as limited RDFS and OWL inference. Virtuoso can be run on both standalone and clustered machines. The standalone triple store server is available through both open source and commercial licensing. We evaluated Virtuoso version 6.4 commercial because we found some bugs in the open source version.

### Data set

**Figure 1:**
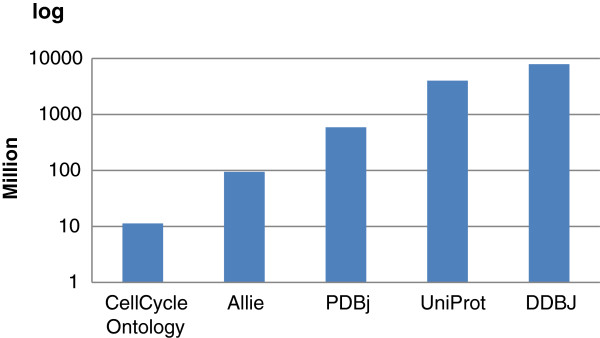
The five biological data sets that were used in our evaluation, with sizes ranging from 11 million to approximately 8 billion triples.

We chose five typical biological data sets to evaluate. The number of triples in these data sets ranging from 10 million to 8 billion. The data were available as either a set of large files, such as uniprot.rdf.gz, uniparc.rdf.gz, and uniref.rdf.gz in the UniProt data set, or a set of small files, e.g., 77,878 files in the PDBj data set. Figure 1 shows the data size for each data set. Their formats and download addresses are as follows: **Cell Cycle Ontology [**[26]**]**:.rdf format, 11,315,866 triples, from http://www.semantic-systems-biology.org/. We downloaded the data on December 21, 2011. **Allie [**[35]*,*[36]**]**:.n3 format, 94,420,989 triples, from ftp://ftp.dbcls.jp/allie/. We used the data published on December 12, 2011. **PDBj [**[37]**]**:.rdf.gz format, 589,987,335 triples, 77,878 files, from ftp://ftp.pdbj.org/XML/rdf/. We downloaded the data on December 19, 2011. **UniProt [**[38]*]*:.rdf.gz format, 4,025,881,829 triples, including 3 larger files, uniprot.rdf.gz, uniparc.rdf.gz, and uniref.rdf.gz, and 7 smaller files, including citations.rdf.gz, enzyme.rdf.gz, journals.rdf.gz, etc. from http://ftp: //ftp.uniprot.org/pub/databases/uniprot/. We used the version that was released in November 2011. **DDBJ [**[39,40]**]**:.rdf.gz format, 7,902,743,055 triples, 330 files, from ftp://ftp.ddbj.nig.ac.jp/ddbj\_database/ ddbj/. We downloaded the data on December 20th, 2011.

### SPARQL Query

The query use cases we used in this study were designed based on the daily usage of the data set. These use cases reflected the main search functions in the website of each data set, http://www.semantic-systems-biology.org/biogateway/queryingfor Cell Cycle Ontology, http://allie.dbcls.jp/ for Allie, http://beta.sparql.uniprot.org/ for UniProt, http://legacy.pdbj.org/index.html for PDBj, and http://www.ddbj.nig.ac.jp/searches-e.html for DDBJ.

The SPARQL queries in our benchmark included queries aimed at retrieving one “record” as well as larger result sets. Our queries included as many as 11 joins. Different types of queries have a large impact on the query store performance. The same query written in two different ways can produce radically different query times. In addition, the designed queries considered the performance of many functions, including join, orderby, filter, distinct, union, optional, count, limit, and offset. Section **Additional file **1**—SPARQL Query** shows the detailed queries that we tested.

### Benchmark

#### Load time

We searched for the best performance for each triple store. We imported the data with default parameters as well as several empirically improved settings and identified the best configuration (please see the section **Additional file **2**—Configuration** for our optimal configuration). We tested each triple store with the best configuration twice and reported the load time as the average cost over the two tests. Every time we cleared the file system memory cache, deleted the previous database and then loaded the data on an empty store.

#### Disk space requirement

The disk space requirement is the total disk storage that is used to load the data set for each triple store. We report the disk space requirement as the size of the whole directory that was used by the data repository.

#### Query response time

We executed the whole query sequence for every triple store and recorded the query response time. We did this five times. Considering some unsteady factor (such as the system cache situation) may incur a higher query response time cost, we removed the highest one and reported the query response time as the average cost of the remaining four queries. In this paper, we present only the average cost; details about the five time costs for each triple store can be found at our website [41].

To evaluate simultaneous executions with multi-clients, we sequentially picked up the queries successfully executed by all the tested triple stores (e.g., we used the 14 queries without case 12, 15, 16, 17, 18 in Cell Cycle Ontology data set) to form five query mixes, and then execute each query mix five times with 1, 4, 8, 64 clients, respectively, for each data set and triple store. We measured their time cost.

#### Query soundness

We checked whether the triple store was able to return query results with the default query setting. For a query that neither gave a result nor provided an error message in one hour, we would report it failed the query. If a query failed, we reported the unsupported clause or error message. In addition, for a query with “limit” predicate, we checked whether the demanding or maximum size was returned. For queries asking for returning all the results, we examined the result size of each triple store. If the result size that some triple store returned was smaller, we tuned its configuration and performed the query again to try to return more results until its maximum results were returned.

### Environment

Our evaluation focused on the data store on a single machine and a single end-user query. We used an Intel(R) Xeon(R) CPU E5649@ 2.53GHz with a 12 core hyperthreaded system (24 virtual cores), 64G of RAM, three 2T SCSI disk storages, an ext3 file system, CentOS release 5.7, and JDK 1.6.0_26.

## Results

### Load time

We conducted performance tuning to determine the best performance for every triple store. We found that Virtuoso having one stream per core to load the data was a good performance point which would keep all parts of the system busy. The results showed that Virtuoso had better performance with parallel loading on a multi-core machine when a set of small files with multiple threads was uploaded. Therefore, to test UniProt loading, we used the Virtuoso procedure language to split all of the files into a set of smaller files that were each composed of 200,000 triples, and we used 12 loading threads in our test. This splitting cost an additional 17 hours of runtime (The last load time for Virtuoso in Table 1 includes this 17 hours). For some triple stores, such as OWLIM-SE, performance was improved by adjusting the JVM parameters (e.g., -Xmx, -Xms, etc.). However, other triple stores, such as Mulgara, were not influenced by adjusting the JVM parameters. For details about our loading approaches, please see our website [41].

**Table 1:** The loading cost for each triple store

**Triple store**	**Cell Cycle**	**Allie**	**PDBj**	**UniProt**	**DDBJ**
	**Ontology**				
OWLIM-SE (min)	3	22	140	3770	7750
Virtuoso (min)	4	47	92	3508	4759
4store (min)	2	12	4834	X	X
Bigdata (min)	3	272	1158	X	X
Mulgara (min)	10	86	X	X	X

**Figure 2:**
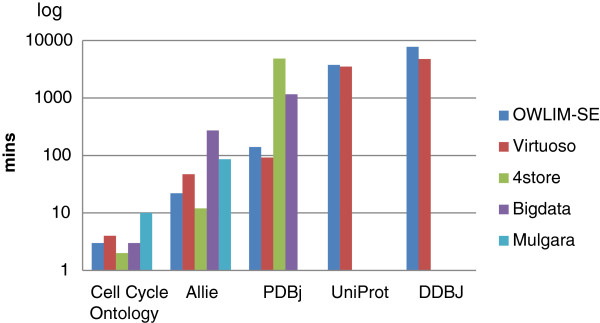
**The loading cost of each triple store.** The loading cost for each triple store for each data set. A missing value indicates that we failed to load the data set.

Table 1 shows that OWLIM-SE and Virtuoso are able to finish all of the loading tasks. The “X” mark in the table indicates a failure to load the data set. Using 4store, the time cost to load approximately 100 million triples in the Allie data set to 500 million triples in the PDBj data set increased 400-fold. Therefore, we did not evaluate the performance of 4store on UniProt or DDBJ because of its poor scalability. Mulgara failed to load PDBj with the error message “Unable to load file: Illegal character ABSA_(A^2)”; “Unexpected XAException” error occurred when loading UniProt and DDBJ. Bigdata had difficulty in loading all of the UniProt data, such that the loading process almost stopped when the loaded triple number exceeded 3.5 billion. However, Bigdata was able to load the data easily when the triple number was less than 3 billion. Figure 2 illustrates the loading cost for each data set.

In addition, the format (such as the data set is composed of a big file or a set of small files) of the data set affected the performance of the triple store. The time to load PDBj, UniProt, and DDBJ was less for Virtuoso compared with OWLIM-SE, while the time to load Cell Cycle Ontology and Allie was greater for Virtuoso than OWLIM-SE. This difference may be partly due to the format of the data; the three former data sets were composed of many small files. The triple number of DDBJ was nearly two times that of UniProt (7.9 billion compared with 4.0 billion, respectively). However, using Virtuoso, the loading cost for DDBJ was much less than two times the loading cost for UniProt. Virtuoso demonstrated good performance when loading multiple small files with multiple threads. Our experiment on OWLIM-SE 4.3 [42] demonstrated that OWLIM-SE 4.3 took less time to load DDBJ compared with UniProt, which also suggests that the format of the data set (e.g., multiple small files) affects the performance of a triple store. The difference can partly come from the data sets themselves since we use five different real data sets.

### Disk space requirement

Table 2 shows the space that was consumed when loading the data set for every triple store. When loading each data set we cleared the database and then loaded the data on an empty store. Therefore the presented space is just what the data set occupied. The experiment shows that the space used by OWLIM-SE increased slowest as the data size increased. 4store, Bigdata and Mulgara were relatively poor.

**Table 2:** The space cost to load the data for each triple store

**Triple store**	**Cell Cycle**	**Allie**	**PDBj**	**UniProt**	**DDBJ**
	**Ontology**				
OWLIM-SE	3.7G	8.2G	27G	213G	513G
Virtuoso	0.84G	6.4G	30G	308G	538G
4store	2.2G	14.7G	66G	X	X
Bigdata	0.78G	6.2G	34G	X	X
Mulgara	2.4G	15.8G	X	X	X

### Query response time and query soundness

#### Cell cycle ontology

**Figure 3:**
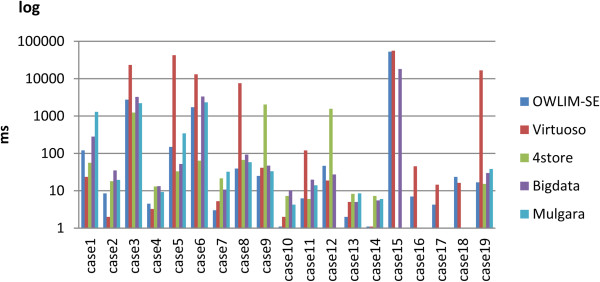
**The results of the Cell Cycle Ontology query evaluation.** The detailed query results for all 19 queries that were submitted to the Cell Cycle Ontology database for each triple store. For queries 16, 17, and 18, only the performance data for OWLIM-SE and Virtuoso are reported because the other triple stores failed to execute these queries.

Table 3 shows the query performance for the Cell Cycle Ontology data set. The “X” mark indicates a query that failed, and boldface shows the fastest response for each query. It is the same to the following tables. Both Virtuoso and OWLIM-SE demonstrated sound query ability. Both Virtuoso and OWLIM-SE completed all of the queries. The query soundness of 4store depended on the setting of the parameter *Softlimit*. In the first query, with *Softlimit* equal to 5000, 4store was able to return all 53 results. However, when *Softlimit* was equal to 1000, 4store returned only 17 results. In addition, 4store, Bigdata and Mulgara could not support the **count()** function in queries 16, 17, and 18. Mulgara returned a zero result for query 15 (the result size should be 7354) and an “Unknown ConstraintExpression exception” for query 12. 4store gave no response to query 15 in one hour.Figure 3 shows the corresponding bar chart for the Cell Cycle Ontology results. For this smallest data set, Virtuoso responded faster than other triple stores for some queries but was slowest for other queries, such as query 5 and query 19. We say that Virtuoso has worst cases. OWLIM-SE performed best on this data set and had no worst cases. Bigdata had average performance on this data set. Although 4store had poor query soundness, the performance of 4store was distinctly better for some cases, such as query 5 and query 6. Mulgara performed the worst of all of the triple stores on this data set.

**Table 3:** The queries for Cell Cycle Ontology

**Endpoint**	**case1**	**case2**	**case3**	**case4**	**case5**	**case6**	**case7**	**case8**	**case9**	**case10**
OWLIM-SE (ms)	121	9	2740	5	149	1722	**3**	**39**	**25**	**1**
Virtuoso (ms)	**24**	**2**	23280	**3**	42500	13073	5	7562	41	2
4store (ms)	56	18	**1236**	13	**33**	**64**	22	67	2035	7
Bigdata (ms)	282	35	3247	13	52	3320	11	93	47	10
Mulgara (ms)	1294	20	2207	9	343	2325	32	58	33	4
**Endpoint**	**case11**	**case12**	**case13**	**case14**	**case15**	**case16**	**case17**	**case18**	**case19**	
OWLIM-SE (ms)	**6**	47	**2**	**1**	52779	**7**	**4**	24	17	
Virtuoso (ms)	120	**19**	5	**1**	56058	46	15	**16**	16721	
4store (ms)	**6**	1563	8	7	X	X	X	X	**15**	
Bigdata (ms)	20	27	5	6	**18126**	X	X	X	30	
Mulgara (ms)	14	X	9	6	X	X	X	X	38	

#### Allie

**Figure 4:**
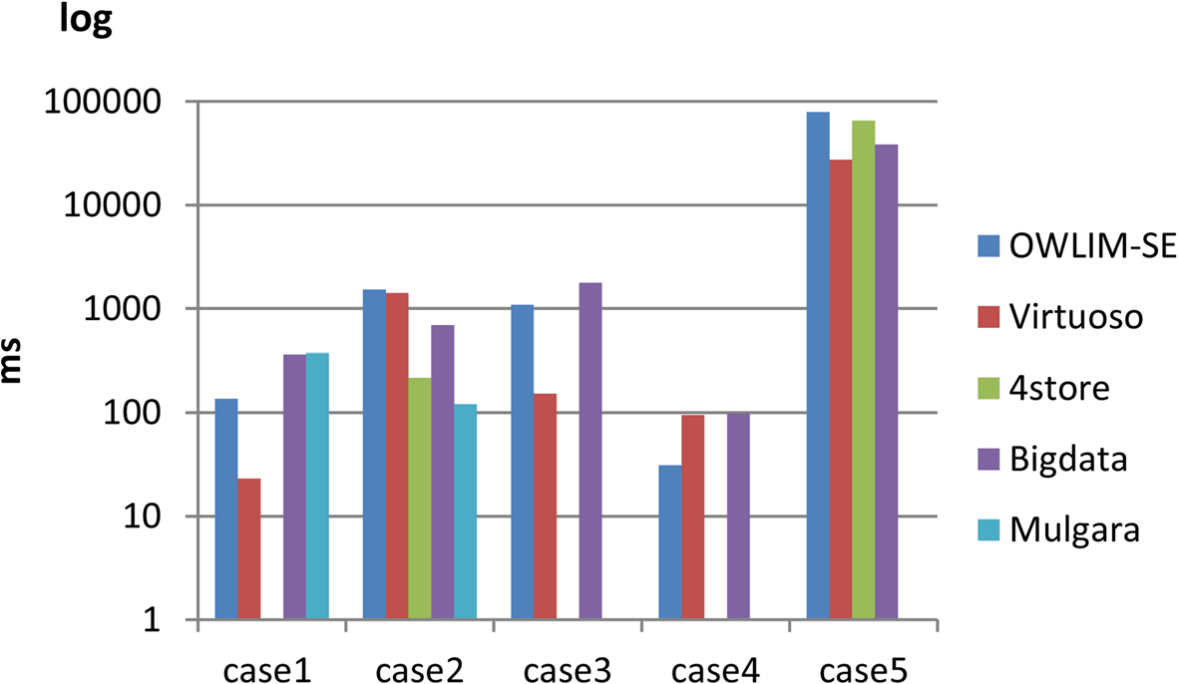
**The results of the Allie query evaluation.** The detailed query results for all five queries that were submitted to the Allie database for each triple store. A missing value indicates that the query failed for that triple store.

Table 4 shows the query performance for the Allie data set. Virtuoso, Bigdata, and OWLIM-SE demonstrated sound query ability on this data set. 4store did not support the **lang()** function in queries 1, 3, and 4. Mulgara was unable to support the arbitrarily complex **ORDER BY** clause.The triple number of this data set nearly 10-fold higher than that of the Cell Cycle Ontology data set. Figure 4 shows that Virtuoso and OWLIM-SE performed better than the other triple stores. For this data set, Virtuoso had no worst cases. Bigdata had average performance on this data set. 4store was limited but performed well on query 2.

**Table 4:** The queries for Allie

**Endpoint**	**case1**	**case2**	**case3**	**case4**	**case5**
OWLIM-SE(ms)	136	1530	1091	**31**	78942
Virtuoso (ms)	**23**	1413	**152**	95	**27299**
4store (ms)	X	217	X	X	65128
Bigdata(ms)	365	690	1779	98	38523
Mulgara(ms)	373	**121**	X	X	X

#### PDBj

**Figure 5:**
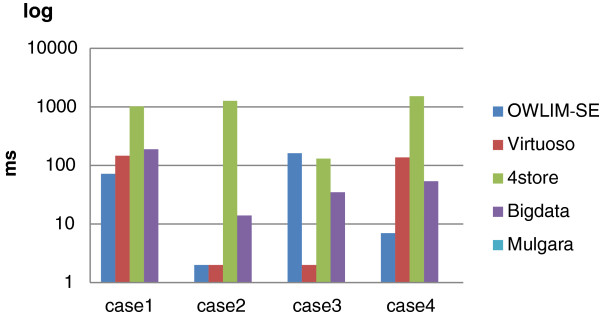
**The results of the PDBj query evaluation.** The detailed query results for all 4 queries that were submitted to the PDBj database for each triple store. The performance of Mulgara is not reported because Mulgara failed to load PDBj.

Virtuoso and OWLIM-SE performed better than the other triple stores on the PDBj data set, as shown in Table 5 and Figure 5. However, neither of them had a significant advantage. 4store demonstrated sound query ability, but the query performance on this data set was the worst of the five triple stores. Bigdata again displayed average performance. Mulgara failed to load the PDBj data set, and therefore we could not present its query performance.

**Table 5:** Query for PDBj

**Endpoint**	**case1**	**case2**	**case3**	**case4**
OWLIM-SE(ms)	**72**	**2**	162	**7**
Virtuoso (ms)	147	**2**	**2**	138
4store (ms)	1025	1274	131	1524
Bigdata(ms)	190	14	35	54
Mulgara(ms)	X	X	X	X

#### UniProt and DDBJ

For the two largest data sets, UniProt and DDBJ, Virtuoso performed the best.

**Figure 6:**
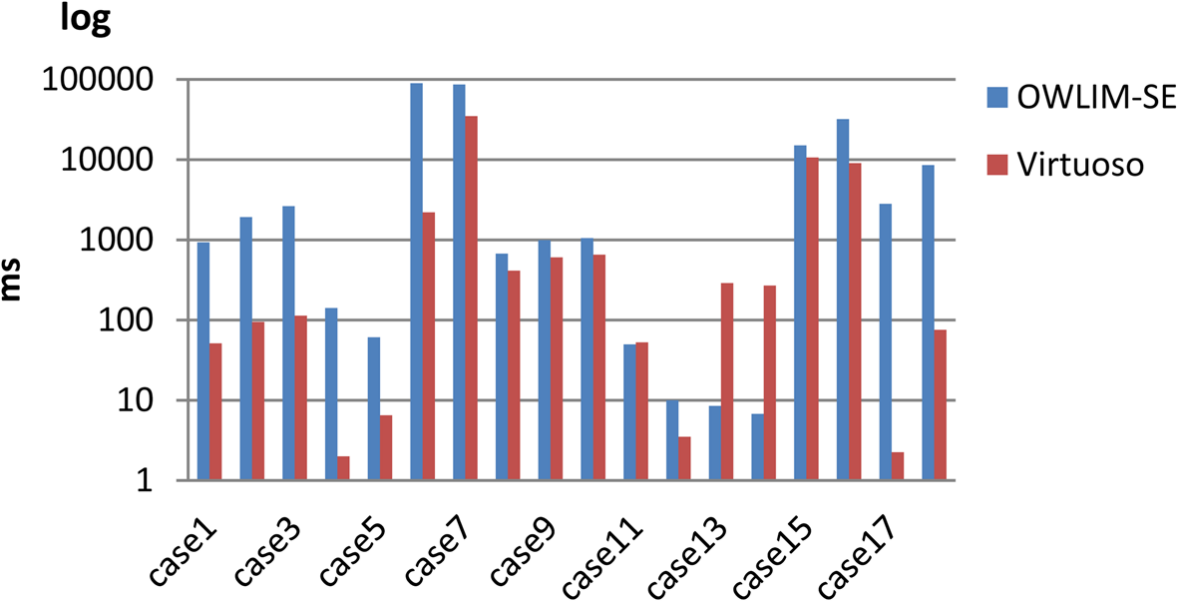
**The results of the UniProt query evaluation.** Only OWLIM-SE and Virtuoso were able to load the UniProt database.

**Figure 7:**
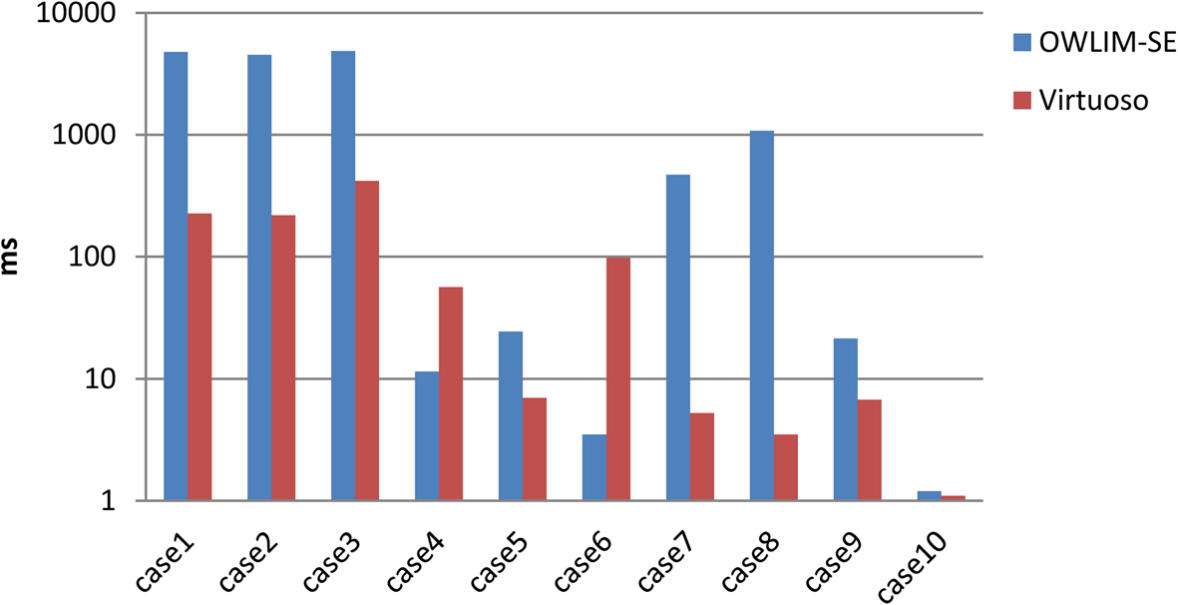
**The results of the DDBJ query evaluation.** Only OWLIM-SE and Virtuoso were able to load the DDBJ database.

We were able to completely load these two data sets only with OWLIM-SE and Virtuoso. Table 6 and Table 7 report that both Virtuoso and OWLIM-SE performed well on the UniProt and DDBJ data sets, respectively. However, Virtuoso performed better as the triple number increased. Figure 6 and Figure 7 are the corresponding bar charts for the results of the UniProt and DDBJ data sets, respectively.

**Table 6:** the queries for UniProt

**Endpoint**	**case1**	**case2**	**case3**	**case4**	**case5**	**case6**	**case7**	**case8**	**case9**	**case10**
OWLIM-SE (ms)	931	1920	2627	142	61	89586	86380	674	994	1053
Virtuoso(ms)	**51**	**95**	**114**	**2**	**7**	**2206**	**34916**	**413**	**605**	**652**
**Endpoint**	**case11**	**case12**	**case13**	**case14**	**case15**	**case16**	**case17**	**case18**		
OWLIM-SE (ms)	**50**	10	**9**	**7**	15037	32055	2818	8548		
Virtuoso (ms)	53	**4**	289	269	**10631**	**9052**	**2**	**76**		

**Table 7:** The queries for DDBJ

**Endpoint**	**case1**	**case2**	**case3**	**case4**	**case5**	**case6**	**case7**	**case8**	**case9**	**case10**
OWLIM-SE (ms)	4783	4528	4867	**12**	25	**4**	470	1078	22	**1**
Virtuoso(ms)	**226**	**218**	**418**	56	**7**	98	**5**	**4**	**7**	**1**

#### Simultaneous execution

Table 8 shows simultaneous executions with multi-clients, 1, 4, 8, and 64 clients respectively. Mulgara reported the error “Interrupted while waiting to acquire lock” when doing queries with over 2 clients. We only evaluated 4store with Cell Cycle Ontology and Allie because it showed unsteady performance with multi-clients when data is larger. Virtuoso, OWLIM-SE and Bigdata finished the simultaneous executions with good scalability.

**Table 8:** Simultaneous execution

**Triple store**	**Number of clients**	**Cell Cycle Ontology**	**Allie**	**PDBj**	**UniProt**	**DDBJ**
OWLIM-SE(ms)	1	6,402	6,704	861	1,651,466	83,179
	4	8,474	13,967	1,041	1,911,144	89,626
	8	14,190	20,891	1,033	2,216,634	109,195
	64	120,126	159,211	2,286	6,058,957	442,181
Virtuoso(ms)	1	14,742	1,421	789	31,876	49,624
	4	22,459	7,189	1,168	50,953	5,246
	8	27,297	9,870	1,655	58,498	10,426
	64	194,850	55,366	8,496	905,697	35,879
4store(ms)	1	4,706	682	x	x	x
	4	15,825	1,413	x	x	x
	8	27,604	2,191	x	x	x
	64	237,246	15,288	x	x	x
Bigdata(ms)	1	10,757	100,683	2,028	x	x
	4	15,617	129,136	2,138	x	x
	8	82,579	850,852	2,051	x	x
	64	108,755	4,467,378	2,930	x	x

## Conclusions

Our paper shows that with appropriate configuration Virtuoso and OWLIM-SE can satisfy the basic requirements to load and query biological data less than 8 billion or so on a single node, for the simultaneous access of 64 clients.

OWLIM-SE performs best for databases with approximately 11 million triples, with no worst query cases; For data sets that contain 94 million and 590 million triples, OWLIM-SE and Virtuoso perform best in the five evaluated triple stores, and they do not show overwhelming advantage over each other; For data over 4 billions Virtuoso works best.

As for other triple stores, (1) 4store performs well on small data sets (e.g. Cell Cycle Ontology) with limited features, and our test shows its scalability is poor; (2) Bigdata demonstrates average performance on both loading and querying and may be a good open source triple store for middle-sized (500 million or so) data set; (3) Mulgara shows a little of fragility.

## Discussion and future work

Our evaluation shows that both Virtuoso and OWLIM-SE are able to efficiently load and query data sets with up to approximately 8 billion triples on a single machine. The scalability of both Virtuoso and OWLIM-SE is good. Virtuoso has the best performance with parallel loading on a multi-core machine for sets of small files with multiple threads. Although Virtuoso has some worst cases when the data set is very small, its performance improves as the number of triples increases. 4store performs the best on small data sets with limited features. The performance of 4store worsens from Cell Cycle Ontology to PDBj as the size of the data set increases, indicating that the scalability of 4store is poor. Bigdata had average performance on all data sets with acceptable loading and query costs. Bigdata may therefore be a good open source triple store for smaller data sets. Because Mulgara failed to load several of the data sets that were tested, its query performance could not be demonstrated.

Our results indicate that 4store can perform well on both loading and querying data with limited features when the number of triples is less than 100 million. For data sets of moderate size (100 million to 500 million), Virtuoso and OWLIM-SE perform similarly. Of the five tested triple stores, Virtuoso performs best on data sets with several billion triples.

The conclusions in our benchmark are basically consistent to BSBM when data size is less than 1 billion, however, not to all other benchmarks. Biological data benchmark [26] shows that OWLIM responded in relatively short time, 4store in moderate time and Virtuoso was slowest. Our benchmark shows some difference. Virtuoso performed best in many cases while it was slowest in some others. Our benchmark proves that Virtuoso had good scalability, while it could perform not well for small data. Another real-world data triple store benchmark [43] shows that Virtuoso was slowest to load the data. Our benchmark shows that Virtuoso worked faster in loading and querying as increasing the data size. In addition our evaluation shows that both Virtuoso and OWLIM-SE scaled well up to 8 billion in both loading and querying on a single node.

Our detailed evaluation of the configurations of each triple store (please see the detailed configurations in Additional file 2 — Configuration for each triple store, or refer to our website) demonstrated that the cost associated with loading the data depends on multiple factors, including the server configuration (e.g., CPU, memory, hard disk, etc.), the system property (e.g., vm.swappiness, JVM, etc.), the application configuration (e.g., cache memory in OWLIM-SE, etc.), and the data format and the size of the data set (e.g., DDBJ is nearly two times the triple size of UniProt, but the loading cost when using Virtuoso is two times less for DDBJ than UniProt, which indicates that the scaling is not proportional, etc.).

For each database, several results were obtained by adjusting parameters that may significantly influence the performance of each triple store. These parameters may also perform differently with different hardware and software platforms as well as with different data sets. A test of all possible parameter combinations is difficult because some data sets, such as UniProt and DDBJ, may take several days to load. Therefore, one limitation of our evaluation is that we cannot guarantee that we have demonstrated the best absolute performance of each triple store.

In the future, we will evaluate federated queries as well as the inference ability of each triple store. The use cases we used in this study were designed based on their daily usage, including do join operations over 10 times, different types of filter operations, and almost all of the clauses that are frequently used in the SPARQL queries. Some other special use cases can be designed to test the detailed performance of each triple store, such as tests of PSO (in predicate-subject-object order) and POS (in predicate-object-subject order) indices. In addition, the triple stores themselves are also improving as newer versions are released. For example, disk space requirements and loading costs have been improved in OWLIM by introducing compression and fixing bugs in the engine. Although Virtuoso 7 seems a mere major update to Virtuoso 6, the underlying technologies are very different. Virtuoso 6 is a row store database, but Virtuoso 7 adopts column store technology, which makes them a totally different performance. For Allie data set, Virtuoso 7 took 7 minutes to import. As for five query use cases, it took 61, 1107, 391, 71 and 5633 milliseconds, respectively. Compared with Virtuoso 6, response for use case 2, 4 and 5 were faster, 1 and 3 were slower. However, we found that there are still some problems to use Virtuoso 7, such as system crashed when uploading our DDBJ data with error log “GPF:Dkpool.c:munmap failed”. We will keep evaluating new triple stores or versions and their clusters, and updating the results in our website http://kiban.dbcls.jp/togordf/wiki.

## Competing interests

The authors declare that they have no competing interests.

## Authors’ contributions

The work presented in this paper was conducted in collaboration between all authors. HW conducted the experiments, analysed the data, and drafted the paper. TF, YY, and JB worked on data collection, use-case design, and software tuning as well as other related tasks. AY coordinated and managed the entire experimental process. All authors have contributed to revisions to the manuscript and have approved the final version of the manuscript.

## Supplementary Material

Additional file 1 **SPARQL Query.** This file includes the details of the SPARQL queries that we used in our evaluations [26]. Click here for file

Additional file 2 **Configuration.** This file presents the modified parameters for each database. Click here for file

## References

[B1] Berners-lee T Hendler J Lassila O **The semantic web-a new form of Web content that is meaningful to computers will unleash a revolution of new possibilities** Sci Am 2001 284 34 43211396337

[B2] **Semantic Web** [http://www.w3.org/2001/sw/]

[B3] Chen H Ding L Wu Z Yu T Dhanapalan L Chen JY **Semantic web for integrated network analysis in biomedicine** Brief Bioinform 2009 10 2 177 192 19304873 10.1093/bib/bbp002

[B4] Cheung KH Smith AK Yip KY Baker CJ Gerstein MB Semantic web approach to database integration in the life sciences 2007 Semantic, Web: Revolutionizing Knowl Discov Life Sci US: Springer 11–30. http://dx.doi.org/10.1007/978-0-387-48438-9_2

[B5] Cantor C **Orchestrating the human genome project** Science 1990 248 49 51 2181666 10.1126/science.2181666

[B6] Benson DA Karsch-Mizrachi I Lipman DJ Ostell J Wheeler DL **GenBank** Nucleic Acids Res 1997 25 1 1 6 9016491 10.1093/nar/25.1.1 PMC146400

[B7] Gollub J Ball CA Binkley G Demeter J Finkelstein DB Hebert JM Hernandez-Boussard T Jin H Kaloper M Matese JC Schroeder M Brown PO Botstein D Sherlock G **The Stanford Microarray Database: data access and quality assessment tools** Nucleic Acids Res 2003 31 1 94 6 12519956 10.1093/nar/gkg078 PMC165525

[B8] Edgar R Domrachev M Lasha AE **Gene expression omnibus: NCBI gene expression and hybridization array data repository** Nucleic Acids Res 2002 30 1 207 110 11752295 10.1093/nar/30.1.207 PMC99122

[B9] Bader GD Betel D Hogue CWV **BIND: the Biomolecular Interaction Network Database** Nucleic Acids Res 2003 31 1 248 250 12519993 10.1093/nar/gkg056 PMC165503

[B10] Desiere F Deutsch EW King NL Nesvizhskii AI Mallick P Eng J Chen S Eddes J Loevenich SN Aebersold R **The PeptideAtlas project** Nucleic Acids Res 2006 34 Database Issue 655 658 10.1093/nar/gkj040 PMC1347403 16381952

[B11] Baralis E Fiori A **Exploring heterogeneous biological data sources**19th International Workshop on Database and Expert Systems Applications 2008 DEXA 647 651 [http://ieeexplore.ieee.org/stamp/stamp.jsp?tp=&arnumber=4624791&isnumber=4624651]

[B12] Martone ME Gupta A Ellisman MH **e-Neuroscience: challenges and triumphs in integrating distributed data from molecules to brains** NatNeuroscience 2004 7 467 472 10.1038/nn1229 15114360

[B13] Chen H Yu T Chen J **Semantic web meets integrative biology: a survey** Brief Bioinform 2013 14 109 125 22492191 10.1093/bib/bbs014

[B14] Antezana E Kuiper M Mironov V **Biological knowledge management: the emerging role of the Semantic Web technologies** Brief Bioinform 2009 10 392 407 19457869 10.1093/bib/bbp024

[B15] Katayama T Wilkinson MD Micklem G Kawashima S Yamaguchi A Nakao M Yamamoto Y Okamoto S Oouchida K Chun HW Aerts J Afzal H Antezana E Arakawa K Aranda B Belleau F Bolleman J Bonnal RJ Chapman B Cock P Eriksson T Gordon P Goto N Hayashi K Horn H Ishiwata R Kaminuma E Kasprzyk A Kawaji H Kido N **The 3rd DBCLS BioHackathon: improving life science data integration with semantic Web technologies** J Biomed Semant 2013 4 6 10.1186/2041-1480-4-6 PMC3598643 23398680

[B16] Consortium TU **Update on activities at the Universal Protein Resource (UniProt) in 2013** Nucleic Acids Res 2013 41 43 47 10.1093/nar/gks1068 PMC3531094 23161681

[B17] Kinjo AR Suzuki H Yamashita R Ikegawa Y Kudou T Igarashi R Kengaku Y Cho H Standley DM Nakagawa A Nakamura H **Protein Data Bank Japan (PDBj): maintaining a structural data archive and resource description framework format** Nucleic Acids Res 2012 40 453 460 10.1093/nar/gkr811 PMC3245181 21976737

[B18] **wwpdb** [http://www.wwpdb.org/]

[B19] Rose PW Bi C Bluhm W Christie CH Dimitropoulos D Dutta S Green RK Goodsell DS Prlic A Quesada M Quinn GB Ramos AG Westbrook JD Young J Zardecki C Berman HM Bourne PE **The RCSB Protein Data Bank: new resources for research and education** Nucleic Acids Res 2013 41 Database-Issue 475 482 10.1093/nar/gks1200 PMC3531086 23193259

[B20] Ulrich EL Akutsu H Doreleijers JF Harano Y Ioannidis YE Lin J Livny M Mading S Maziuk D Miller Z Nakatani E Schulte CF Tolmie DE Kent Wenger R Yao H Markley JL **BioMagResBank** Nucleic Acids Res 2008 36 Database issue D402 D408 [http://dx.doi.org/10.1093/nar/gkm957] 17984079 10.1093/nar/gkm957 PMC2238925

[B21] Velankar S Alhroub Y Best C Caboche S Conroy MJ Dana JM Montecelo MAF van Ginkel G Golovin A Gore SP Gutmanas A Haslam P Hendrickx PMS Heuson E Hirshberg M John M Lagerstedt I Mir S Newman LE Oldfield T Patwardhan A Rinaldi L Sahni G Sanz-García E Sen S Slowley R Suarez-Uruena A Swaminathan GJ Symmons MF Vranken WF **PDBe: Protein Data Bank in Europe** Nucleic Acids Res 2012 40 Database-Issue 445 452 10.1093/nar/gkr998 PMC3245096 22110033

[B22] Rusher J **TripleStore** Semantic Web Advanced Development for Europe (SWAD-Europe), Workshop on Semantic Web Storage and Retrieval [http://www.w3.org/2001/sw/Europe/events/20031113-storage/]

[B23] Guo Y Pan Z Heflin J **LUBM: A benchmark for OWL knowledge base systems** Web Semant Sci Serv Agents World Wide Web 2005 3 158 182

[B24] **BSBM V3 Results (February 2011)** [http://www4.wiwiss.fu-berlin.de/bizer/BerlinSPARQLBenchmark/results/V6/index.html]

[B25] Schmidt M Hornung T Lausen G Pinkel C SP2Bench: a SPARQL performance benchmark 2008 Berlin: Springer

[B26] Mironov V Seethappan N Blondé W Antezana E Splendiani A Kuiper M **Gauging triple stores with actual biological data** BMC bioinformatics 2012 13 S3 22373359 10.1186/1471-2105-13-S1-S3 PMC3471352

[B27] **4store** [http://4store.org/]

[B28] Harris S Lamb N Shadbolt N **4store: the design and implementation of a clustered rdf store**5th International Workshop on Scalable Semantic Web Knowledge Base Systems (SSWS2009) [http://ceur-ws.org/Vol-517/SSWS09-Proceedings.pdf], 2009

[B29] **bigdata** [http://www.systap.com/bigdata.htm]

[B30] **ontotext** [http://www.ontotext.com/owlim]

[B31] Kiryakov A Ognyanov D Manov D OWLIM – a Pragmatic Semantic Repository for OWL 2005 Berlin: Springer

[B32] **mulgara SEMANTIC STORE** [http://www.mulgara.org/]

[B33] **OPENLINK SOFTWARE** [http://virtuoso.openlinksw.com/]

[B34] Erling O Mikhailov I **RDF support in the virtuoso DBMS** Proceedings of the 1st Conference on Social Semantic Web CSSW 2007 Berlin: Springer

[B35] Yamamoto Y Yamaguchi A Bono H Takagi T **Allie: a database and a search service of abbreviations and long forms** Database 2011 [http://dx.doi.org/10.1093/database/bar013] 10.1093/database/bar013 PMC3077826 21498548

[B36] **Allie: A Search Service for Abbreviation/Long Form** [http://allie.dbcls.jp/]

[B37] **PDBj** [http://www.pdbj.org/]

[B38] **UniProt** [http://www.uniprot.org/]

[B39] Kodama Y Mashima J Kaminuma E Gojobori T Ogasawara O Takagi T Okubo K Nakamura Y **The DNA Data Bank of Japan launches a new resource, the DDBJ omics archive of functional genomics experiments** Nucleic Acids Res 2012 40 Database-Issue 38 42 10.1093/nar/gkr994 PMC3244990 22110025

[B40] **DDBJ:DNA Data Bank of Japan** [http://www.ddbj.nig.ac.jp/]

[B41] **RDF for life science** [http://kiban.dbcls.jp/togordf/]

[B42] **RDF for life science** [http://kiban.dbcls.jp/togordf/wiki/OwlimSe4.3]

[B43] Voigt M Mitschick A Schulz J **Yet another triple store benchmark? Practical experiences with real-world data** Semantic Digital Archives 2012 85 94 [http://ceur-ws.org/Vol-912/paper7.pdf]

