# Supplementary material for: BioBenchmark Toyama 2012: an evaluation of the performance of triple stores on biological data
Source: J Biomed Semantics. 2014 Jul 10;5:32. doi: 10.1186/2041-1480-5-32 (PMC4118313; doi:10.1186/2041-1480-5-32)
Supplement: Additional file 1 — SPARQL Query. This file includes the details of the SPARQL queries that we used in our evaluations [26]. [file 2041-1480-5-32-S1.pdf]

Cell Cycle Ontology [26]:

```
#case1
PREFIX rdfs:<http://www.w3.org/2000/01/rdf-schema#>
PREFIX ssb:<http://www.semantic-systems-biology.org/SSB#>
PREFIX cco_tc:<http://www.semantic-systems-biology.org/cco_tc#>
SELECT distinct ?protein ?protein_id
WHERE {
  ?protein_id   ssb:has_function    ?function_id.
  ?function_id  ssb:is_a            cco_tc:CCO_F0000031.
  ?protein_id   ssb:located_in      ?location_id.
  ?location_id  ssb:is_a            cco_tc:CCO_C0000252.
  ?protein_id   ssb:participates_in ?process_id.
  ?process_id   ssb:is_a            cco_tc:CCO_P0000117.
  ?protein_id   rdfs:label          ?protein.
}

#case2
PREFIX rdfs:<http://www.w3.org/2000/01/rdf-schema#>
PREFIX ssb:<http://www.semantic-systems-biology.org/SSB#>
PREFIX term_id: <http://www.semantic-systems-biology.org/cco#CCO_B0000007>
SELECT ?function ?participates_in ?located_in
WHERE {
{
  term_id: ssb:has_function ?f.
  ?f rdfs:label ?function.
}
UNION
{
 term_id: ssb:participates_in ?p.
  ?p rdfs:label ?participates_in.
}
UNION
{
  term_id: ssb:located_in ?l.
  ?l rdfs:label ?located_in.
}
}

#case3
PREFIX rdf: <http://www.w3.org/1999/02/22-rdf-syntax-ns#>
PREFIX rdfs:<http://www.w3.org/2000/01/rdf-schema#>
PREFIX ssb:<http://www.semantic-systems-biology.org/SSB#>
SELECT distinct  ?protein_name ?definition ?interaction_name ?IntAct_id
WHERE {
 ?protein_id rdf:type        ssb:protein.
 ?protein_id ssb:Definition  ?Def.
 ?Def        ssb:def         ?definition.
 FILTER regex(?definition,   'Breast cancer', 'i').
 ?protein_id rdfs:label      ?protein_name.
 ?protein_id  ssb:participates_in   ?interaction.
 ?interaction rdf:type              ssb:interaction.
  ?interaction rdfs:label            ?interaction_name.
 ?interaction ssb:xref              ?xref.
 ?xref        ssb:acc               ?IntAct_id
 }
}

#case4
PREFIX rdfs:<http://www.w3.org/2000/01/rdf-schema#>
PREFIX ssb:<http://www.semantic-systems-biology.org/SSB#>
PREFIX cco:<http://www.semantic-systems-biology.org/cco#>
SELECT ?description ?transformed_protein_name ?cco_id
WHERE {
  cco:CCO_B0001575  ssb:transforms_into    ?cco_id.
   ?cco_id      ssb:Definition        ?Def.
   ?Def         ssb:def               ?description.
   ?cco_id      rdfs:label            ?transformed_protein_name.
}

#case5
PREFIX rdfs:<http://www.w3.org/2000/01/rdf-schema#>
PREFIX ssb:<http://www.semantic-systems-biology.org/SSB#>
PREFIX cco_S_pombe:<http://www.semantic-systems-biology.org/cco_S_pombe#>
SELECT ?prot_label ?biological_process_label
WHERE {
```

```
 ?prot               ssb:is_a             cco_S_pombe:CCO_B0000000 .
 ?prot               rdfs:label          ?prot_label .
 ?prot               ssb:participates_in  ?biological_process .
 ?biological_process rdfs:label          ?biological_process_label
}

#case6
PREFIX rdfs:<http://www.w3.org/2000/01/rdf-schema#>
SELECT ?term_id ?term_name
WHERE {
 ?term_id     rdfs:label    ?term_name.
 filter regex(str(?term_name), 'cell cycle')
}

#case7
PREFIX rdfs:<http://www.w3.org/2000/01/rdf-schema#>
PREFIX ssb:<http://www.semantic-systems-biology.org/SSB#>
PREFIX cco_S_pombe_tc:<http://www.semantic-systems-biology.org/cco_S_pombe_tc#>
SELECT distinct ?protein
WHERE {
  ?protein_id       ssb:is_a         cco_S_pombe_tc:CCO_B0000000.
  ?protein_id       ssb:located_in   ?location_id.
  ?location_id      ssb:is_a         cco_S_pombe_tc:CCO_C0000239.
  ?protein_id       rdfs:label       ?protein.
}

#case8
PREFIX rdfs:<http://www.w3.org/2000/01/rdf-schema#>
PREFIX ssb:<http://www.semantic-systems-biology.org/SSB#>
PREFIX cco_A_thaliana:<http://www.semantic-systems-biology.org/cco_A_thaliana#>
SELECT ?uniprot_name ?AT_code
WHERE {
  ?prot ssb:is_a        cco_A_thaliana:CCO_B0000000 .
  ?prot rdfs:label     ?uniprot_name .
  ?prot ssb:encoded_by  ?gene .
  ?gene ssb:xref        ?b .
  ?b    ssb:dbname      ?database .
  ?b    ssb:acc         ?AT_code .
  FILTER(?database = 'TAIR')
}

#case9
PREFIX rdf: <http://www.w3.org/1999/02/22-rdf-syntax-ns#>
PREFIX rdfs:<http://www.w3.org/2000/01/rdf-schema#>
PREFIX ssb:<http://www.semantic-systems-biology.org/SSB#>
PREFIX cco_A_thaliana_tc:<http://www.semantic-systems-biology.org/cco_A_thaliana_tc#>
SELECT distinct ?protein_name ?function
WHERE {
 ?protein_id      rdf:type            ssb:protein.
 ?protein_id      ssb:is_a            cco_A_thaliana_tc:CCO_B0000000.
 ?protein_id      ssb:has_function    ?subfunction_id.
 ?subfunction_id  ssb:is_a            ?function_id.
 ?protein_id      ssb:located_in      ?location_id.
 ?location_id     ssb:is_a            cco_A_thaliana_tc:CCO_C0000324.
 ?function_id     ssb:Definition      ?def.
 ?def             ssb:def             ?function.
 ?protein_id      rdfs:label          ?protein_name.
 FILTER regex(?function, 'hydrolysis','i').
}

#case10
PREFIX rdfs:<http://www.w3.org/2000/01/rdf-schema#>
PREFIX ssb:<http://www.semantic-systems-biology.org/SSB#>
PREFIX cco:<http://www.semantic-systems-biology.org/cco#>
SELECT ?term_label
WHERE {
   cco:CCO_P0000003 rdfs:label  ?term_label.
}

#case11
PREFIX rdfs:<http://www.w3.org/2000/01/rdf-schema#>
PREFIX term_id: <http://www.semantic-systems-biology.org/cco_A_thaliana#CCO_B0002337>

SELECT ?term_as_child ?outwardarrow ?head_name ?tail_name ?inwardarrow ?term_as_parent
WHERE {
  {
```

```
  term_id:       ?outwardarrow   ?head_id.
  term_id:       rdfs:label      ?term_as_child.
#  ?outwardarrow  rdfs:label      ?outward_arrow.
  ?head_id       rdfs:label      ?head_name.
  }
UNION{
  ?tail_id       ?inwardarrow    term_id:.
  ?tail_id       rdfs:label      ?tail_name.
#  ?inwardarrow   rdfs:label      ?inward_arrow.
  term_id:       rdfs:label      ?term_as_parent.
  }
}

#case12
PREFIX rdfs:<http://www.w3.org/2000/01/rdf-schema#>
PREFIX ssb:<http://www.semantic-systems-biology.org/SSB#>
PREFIX term_id: <http://www.semantic-systems-biology.org/cco#CCO_B0001733>
SELECT distinct ?name ?definition ?db ?nr ?organism ?comment ?synonym ?scope ?syn_db ?syn_nr ?xref_db ?xref_nr ?subnamespace ?alt_id
WHERE {
  {term_id: rdfs:label ?name}
UNION{
  term_id: ssb:Definition ?a.
    {?a ssb:def ?definition.}
  UNION{
      ?a ssb:DbXref ?b.
      ?b ssb:dbname ?db.
      ?b ssb:acc ?nr.
      }
    }
  }
UNION
{
term_id: ?has_source ?organism_id.
?organism_id a ssb:taxon.
?organism_id rdfs:label ?organism.
}
UNION
  {term_id: rdfs:comment ?comment}
UNION{
  term_id: ssb:synonym ?a.
  ?a ssb:syn ?synonym.
    ?a ssb:DbXref ?b.
    ?b ssb:dbname ?syn_db.
    ?b ssb:acc ?syn_nr.
    }
  }
UNION{
  term_id: ssb:xref ?a.
  ?a ssb:dbname ?xref_db.
  ?a ssb:acc ?xref_nr.
  }
UNION{
  term_id: a ?subnamespace
  }
UNION{
  term_id: ssb:hasAlternativeId ?alt_id.
  }
}

#case13
PREFIX rdfs:<http://www.w3.org/2000/01/rdf-schema#>
PREFIX ssb:<http://www.semantic-systems-biology.org/SSB#>
PREFIX cco:<http://www.semantic-systems-biology.org/cco#>
SELECT ?name ?CCO_id
WHERE {
  ?CCO_id   ssb:is_a     cco:CCO_P0000160.
  ?CCO_id   rdfs:label   ?name.
}

#case14
PREFIX rdfs:<http://www.w3.org/2000/01/rdf-schema#>
PREFIX ssb:<http://www.semantic-systems-biology.org/SSB#>
PREFIX cco:<http://www.semantic-systems-biology.org/cco#>
SELECT ?name_of_parent ?CCO_id
WHERE {
  cco:CCO_F0001848    ssb:is_a     ?CCO_id.
```

```
  ?CCO_id             rdfs:label   ?name_of_parent.
}

#case15
PREFIX rdfs:<http://www.w3.org/2000/01/rdf-schema#>
PREFIX ssb:<http://www.semantic-systems-biology.org/SS
SELECT ?name ?found_in ?type_of_found_text
WHERE {
  FILTER regex(str(?found_in), 'cell', 'i')
  FILTER regex(str(?found_in), 'cycle', 'i')
  ?term_id rdfs:label ?name.
  {
   ?term_id ?type_of_found_text ?found_in.
   ?term_id rdfs:label ?found_in.
  }
  UNION
  {
   ?term_id ?type_of_found_text ?a.
   ?term_id ssb:Definition ?a.
   ?a ssb:def ?found_in.
  }
  UNION
  {
   ?term_id ?type_of_found_text ?a.
   ?term_id ssb:synonym ?a.
   ?a ssb:syn ?found_in.
  }
  UNION
  {
   ?term_id ?type_of_found_text ?found_in.
   ?term_id rdfs:comment ?found_in.
  }
 }
ORDER BY ?term_id

#case16
PREFIX ssb:<http://www.semantic-systems-biology.org/SSB#>
PREFIX cco_A_thaliana:<http://www.semantic-systems-biology.org/cco_A_thaliana#>
SELECT distinct count(?term_id)
WHERE {
 {
   ?term_id ssb:is_a cco_A_thaliana:CCO_B0000000.
 }
 UNION
 {
   ?term_id ssb:is_a cco_A_thaliana:CCO_U0000011.
 }
 UNION
 {
   ?term_id ssb:is_a cco_A_thaliana:CCO_U0000007.
 }
 ?term_id    ssb:has_source  cco_A_thaliana:CCO_T0000034 .
}

#case17
PREFIX ssb:<http://www.semantic-systems-biology.org/SSB#>
PREFIX cco_S_pombe:<http://www.semantic-systems-biology.org/cco_S_pombe#>
SELECT distinct count(?term_id)
WHERE {
  ?term_id ssb:is_a cco_S_pombe:CCO_U0000008.
}

#case18
PREFIX ssb:<http://www.semantic-systems-biology.org/SSB#>
PREFIX cco:<http://www.semantic-systems-biology.org/cco#>
SELECT distinct count(?term_id)
WHERE {
  ?term_id ssb:is_a cco:CCO_U0000008.
  ?term_id ssb:has_source cco:CCO_T0000017.
}

#case19
PREFIX rdf: <http://www.w3.org/1999/02/22-rdf-syntax-ns#>
PREFIX rdfs:<http://www.w3.org/2000/01/rdf-schema#>
PREFIX ssb:<http://www.semantic-systems-biology.org/SSB#>
PREFIX cco_A_thaliana:<http://www.semantic-systems-biology.org/cco_A_thaliana#>
```

```
SELECT ?protein ?participates_in_interaction
WHERE {
  ?term_id      ssb:has_source        cco_A_thaliana:CCO_T0000034 .
  ?term_id      ssb:participates_in   ?interaction.
  ?interaction  rdf:type             ssb:interaction.
  ?term_id      rdfs:label           ?protein.
  ?interaction  rdfs:label           ?participates_in_interaction.
}
```

Allie:

```
#case1
PREFIX rdfs: <http://www.w3.org/2000/01/rdf-schema#>
PREFIX allie: <http://purl.org/allie/ontology/201108#>
select distinct ?X ?Y
where {
[ allie:inResearchAreaOf ?X;
allie:contains [
allie:hasMemberOf [
allie:hasShortFormOf [
rdfs:label "SPF"@EN ;
allie:hasLongFormOf [
rdfs:label ?Y ;
.
FILTER ( lang(?Y) = "en" )}

#case2
PREFIX rdfs: <http://www.w3.org/2000/01/rdf-schema#>
PREFIX allie: <http://purl.org/allie/ontology/201108#>
select distinct ?X ?Y ?Z
where {
[ allie:inResearchAreaOf ?X;
allie:contains [
allie:hasMemberOf [
allie:hasShortFormOf [
  rdfs:label "SPF"@EN ;
allie:hasLongFormOf [
  rdfs:label ?Y ;
;
allie:appearsIn [
allie:hasMemberOf ?Z .
}

#case3
PREFIX rdfs: <http://www.w3.org/2000/01/rdf-schema#>
PREFIX rdf: <http://www.w3.org/1999/02/22-rdf-syntax-ns#>
PREFIX xsd: <http://www.w3.org/2001/XMLSchema#>
PREFIX mesh: <http://www.nlm.nih.gov/mesh/2011#>
PREFIX allie: <http://purl.org/allie/ontology/201108#>
select ?freq ?SF ?LF
where {
  [ a allie:PairCluster;
  allie:inResearchAreaOf mesh:D001432;
  allie:hasShortFormRepresentationOf [ rdfs:label ?SF ;
  allie:hasLongFormRepresentationOf [ rdfs:label ?LF ;
  allie:frequency ?freq.
FILTER ( lang(?SF) = "en" && lang(?LF) = "en" && xsd:integer(?freq))
}
ORDER BY DESC (xsd:integer(?freq)) ?SF ?LF;

#case4
PREFIX rdfs: <http://www.w3.org/2000/01/rdf-schema#>
PREFIX rdf: <http://www.w3.org/1999/02/22-rdf-syntax-ns#>
PREFIX xsd: <http://www.w3.org/2001/XMLSchema#>
PREFIX allie: <http://purl.org/allie/ontology/201108#>
select distinct ?F ?lfr ?L ?lf
where {
[ a allie:PairCluster;
allie:contains [
allie:hasMemberOf [
allie:hasLongFormOf [
  rdfs:label ?lf;
  allie:frequency ?lfr ;
;
allie:frequency ?F;
```

```
allie:hasShortFormRepresentationOf [ rdfs:label "SPF"@EN ;
allie:hasLongFormRepresentationOf [ rdfs:label ?L .
FILTER ( lang(?L) = "en" && lang(?lf) = "en" )
}
ORDER BY DESC(xsd:integer(?F)) DESC(xsd:integer(?lfr))

#case5
PREFIX rdfs: <http://www.w3.org/2000/01/rdf-schema#>
PREFIX rdf: <http://www.w3.org/1999/02/22-rdf-syntax-ns#>
PREFIX xsd: <http://www.w3.org/2001/XMLSchema#>
PREFIX allie: <http://purl.org/allie/ontology/201108#>
select ?SF ?LF ?freq
where {
[ a allie:PairCluster;
allie:frequency ?freq;
allie:contains [
allie:hasMemberOf [
  allie:hasShortFormOf [
    rdfs:label ?SF ;
  allie:hasLongFormOf [
    rdfs:label ?LF;
.
FILTER (xsd:integer(?freq) > 10
  && !regex(str(?SF), "^s", "i")
  && regex(str(?LF), "^specific", "i"))
}
ORDER BY DESC (xsd:integer(?freq));
```

PDBj:

```
#case1
prefix pdbjrdf: <http://pdbj.org/rdf/>
prefix pdbjschema:<http://pdbj.org/schema/pdbx-v40.owl#>
select ?descriptor ?title ?struct_keywords ?gene_src_scientific_name ?authorname
from <http://pdbj.dbcls.jp>
where {
?entry  pdbjschema:entry.id     "107L" ;
                pdbjschema:referenced_by_struct ?structure;
                pdbjschema:referenced_by_struct_keywords  ?struct_keywords;
                pdbjschema:of_datablock                   ?of_datablock .
?structure    pdbjschema:struct.pdbx_descriptor ?descriptor;
              pdbjschema:struct.title ?title .
?of_datablock  pdbjschema:has_entity_src_genCategory ?entity_src_genCategory;
               pdbjschema:has_audit_authorCategory ?authorCategory.
?entity_src_genCategory pdbjschema:has_entity_src_gen ?entity_src_gen.
?entity_src_gen  pdbjschema:entity_src_gen.pdbx_gene_src_scientific_name ?gene_src_scientific_name.
?authorCategory  pdbjschema:has_audit_author ?audit_author.
?audit_author    pdbjschema:audit_author.name ?authorname .
}

#case2
prefix pdbjrdf: <http://pdbj.org/rdf/>
prefix pdbjschema:<http://pdbj.org/schema/pdbx-v40.owl#>
select  ?authorname
from <http://pdbj.dbcls.jp>
where {
pdbjrdf:107L    pdbjschema:has_audit_authorCategory ?authorCategory.
?authorCategory  pdbjschema:has_audit_author ?audit_author.
?audit_author    pdbjschema:audit_author.name ?authorname .
}

#case3
prefix pdbjrdf: <http://pdbj.org/rdf/>
prefix pdbjschema:<http://pdbj.org/schema/pdbx-v40.owl#>
select ?citation ?predicate ?contents
from <http://pdbj.dbcls.jp>
where {
<http://pdbj.org/rdf/107L>     pdbjschema:has_citationCategory  ?citationCategory.
?citationCategory pdbjschema:has_citation ?citation.
 ?citation ?predicate ?contents
}

#case4
prefix pdbjrdf: <http://pdbj.org/rdf/>
prefix pdbjschema:<http://pdbj.org/schema/pdbx-v40.owl#>
```

```
select ?has_entity ?entitypdbx_description  ?referenced_by_entity_src_gen
?entitytype ?entitypdbx_number_of_molecules ?entity_src_gen_gene_src_species
from <http://pdbj.dbcls.jp>
where {
pdbjrdf:107L     pdbjschema:has_entityCategory  ?has_entityCategory.
?has_entityCategory pdbjschema:has_entity ?has_entity .
?has_entity         pdbjschema:entity.pdbx_description ?entitypdbx_description;
pdbjschema:entity.type ?entitytype;pdbjschema:entity.pdbx_number_of_molecules ?entitypdbx_number_of_molecules.
?referenced_by_entity_src_gen pdbjschema:entity_src_gen.gene_src_species ?entity_src_gen_gene_src_species}
}
```

UniProt:

```
#case1
PREFIX :<http://purl.uniprot.org/core/> SELECT ?x  WHERE { ?x a :Taxon . } limit 300 offset 100000

#case2
PREFIX :<http://purl.uniprot.org/core/> SELECT ?x  WHERE { ?x a :Taxon . } limit 300 offset 200000

#case3
PREFIX :<http://purl.uniprot.org/core/> SELECT ?x WHERE { ?x a :Taxon . } limit 300 offset 300000

#case4
PREFIX :<http://purl.uniprot.org/core/> PREFIX rdfs:<http://www.w3.org/2000/01/rdf-schema#>
SELECT ?x ?y WHERE  { ?x a :Taxon . ?x :scientificName ?y . ?x rdfs:subClassOf <http://purl.uniprot.org/taxonomy/2> }
#case5
PREFIX :<http://purl.uniprot.org/core/> SELECT ?protein WHERE { ?protein a :Protein . ?protein :mnemonic "A4_HUMAN" }

#case6
PREFIX :<http://purl.uniprot.org/core/>  PREFIX rdfs:<http://www.w3.org/2000/01/rdf-schema#>
PREFIX db:<http://purl.uniprot.org/database/>
SELECT ?protein  WHERE { ?protein a :Protein . ?protein rdfs:seeAlso ?db . ?db :database db:PDB }

#case7
PREFIX :<http://purl.uniprot.org/core/> PREFIX skos:<http://www.w3.org/2004/02/skos/core#>
SELECT ?protein ?name
WHERE {
?protein a :Protein .
?protein :recommendedName ?nameH .
?nameH :fullName ?name .
?protein :encodedBy ?gene .
?gene skos:prefLabel ?text .
FILTER REGEX(?text, "DNA") }

#case8
PREFIX :<http://purl.uniprot.org/core/>
SELECT ?protein ?begin ?end
WHERE {
?protein a :Protein .
?protein :annotation ?annotation .
?annotation a :Transmembrane_Annotation .
?annotation :range ?range .
?range :begin ?begin .
?range :end ?end } limit 200 offset 20000

#case9
PREFIX :<http://purl.uniprot.org/core/>
SELECT ?protein ?begin ?end
WHERE {
?protein a :Protein .
?protein :annotation ?annotation .
?annotation a :Transmembrane_Annotation .
?annotation :range ?range .
?range :begin ?begin .
?range :end ?end } limit 200 offset 30000

#case10
PREFIX : <http://purl.uniprot.org/core/> PREFIX rdfs: <http://www.w3.org/2000/01/rdf-schema#>
SELECT ?related
WHERE {
?protein a :Protein .
?protein :classifiedWith <http://purl.uniprot.org/keywords/3> .
?protein rdfs:seeAlso ?related }

#case11
```

```
PREFIX :<http://purl.uniprot.org/core/> PREFIX rdfs:<http://www.w3.org/2000/01/rdf-schema#>
PREFIX skos:<http://www.w3.org/2004/02/skos/core#>
SELECT ?name ?text
WHERE {
?protein a :Protein .
?protein :encodedBy ?gene .
?gene skos:prefLabel ?name .
?protein :organism <http://purl.uniprot.org/taxonomy/9606> .
?protein :annotation ?annotation .
?annotation a :Disease_Annotation .
?annotation rdfs:comment ?text } limit 300

#case12
PREFIX :<http://purl.uniprot.org/core/> PREFIX rdf:<http://www.w3.org/1999/02/22-rdf-syntax-ns#>
PREFIX rdfs:<http://www.w3.org/2000/01/rdf-schema#>
SELECT ?protein ?aa
WHERE {
?protein a :Protein .
?protein :organism ?organism.
?organism rdfs:subClassOf <http://purl.uniprot.org/taxonomy/2> .
?protein :sequence ?s .
?s rdf:value ?aa . } limit 200

#case13
PREFIX :<http://purl.uniprot.org/core/>
SELECT ?protein ?x WHERE { ?protein a :Protein . ?protein :modified "2011-11-16". } limit 200

#case14
PREFIX :<http://purl.uniprot.org/core/> ASK WHERE { ?protein a :Protein . ?protein :modified "2011-11-16". }

#case15
PREFIX :<http://purl.uniprot.org/core/> PREFIX taxon:<http://purl.uniprot.org/taxonomy/>
CONSTRUCT {?protein a :HumanProtein .} WHERE { ?protein a :Protein . ?protein :organism taxon:9606 . }

#case16
PREFIX :<http://purl.uniprot.org/core/> CONSTRUCT {?x a :Concept . } WHERE { ?x a :Taxon . }

#case17
DESCRIBE http://purl.uniprot.org/embl-cds/AAO89367.1

#case18
PREFIX taxon:<http://purl.uniprot.org/taxonomy/> DESCRIBE taxon:9606 FROM <http://purl.uniprot.org/taxonomy/>
```

DDBJ:

```
#case1
select distinct ?s  where{
 ?s <http://sabi.ddbj.nig.ac.jp/core/item/feature> ?b .
 ?s <http://sabi.ddbj.nig.ac.jp/core/item/moltype> "DNA" .
 ?b <http://sabi.ddbj.nig.ac.jp/core/qualifier/db_xref_taxon>  <http://www.ncbi.nlm.nih.gov/taxonomy/7955> .
} offset 20000 limit 100

#case2
select distinct ?s  where{
 ?s <http://sabi.ddbj.nig.ac.jp/core/item/feature> ?b .
 ?s <http://sabi.ddbj.nig.ac.jp/core/item/moltype> "DNA" .
 ?b <http://sabi.ddbj.nig.ac.jp/core/qualifier/db_xref_taxon>  <http://www.ncbi.nlm.nih.gov/taxonomy/7955> .
} offset 20100 limit 100

#case3
select distinct ?s  where{
 ?s <http://sabi.ddbj.nig.ac.jp/core/item/feature> ?b .
 ?s <http://sabi.ddbj.nig.ac.jp/core/item/moltype> "DNA" .
 ?b <http://sabi.ddbj.nig.ac.jp/core/qualifier/db_xref_taxon>  <http://www.ncbi.nlm.nih.gov/taxonomy/7955> .
} offset 40000 limit 100

#case4
prefix qualifier:<http://sabi.ddbj.nig.ac.jp/core/qualifier/>
select ?source ?mRNA ?clone_lib ?mol_type ?db_xref_taxon ?dev_stage
where{
<http://sabi.ddbj.nig.ac.jp/ddbj/data/CR760162> <http://sabi.ddbj.nig.ac.jp/core/item/feature> ?source.
?source qualifier:clone  ?clone;
          qualifier:clone_lib    ?clone_lib;
          qualifier:mol_type     ?mol_type;
          qualifier:db_xref_taxon ?db_xref_taxon;
```

```
          qualifier:dev_stage    ?dev_stage.
}

#case5
prefix qualifier:<http://sabi.ddbj.nig.ac.jp/core/qualifier/>
select distinct ?mRNA ?location ?gene ?locus_tag ?product
where{
<http://sabi.ddbj.nig.ac.jp/ddbj/data/CR760162> <http://sabi.ddbj.nig.ac.jp/core/item/feature> ?mRNA .
?mRNA qualifier:location         ?location;
qualifier:gene ?gene;
qualifier:locus_tag      ?locus_tag;
qualifier:product        ?product.
}

#case6
prefix qualifier:<http://sabi.ddbj.nig.ac.jp/core/qualifier/>
select ?CDS ?gene ?locus_tag  ?product ?note ?db_xref
where{
<http://sabi.ddbj.nig.ac.jp/ddbj/data/CR760162> <http://sabi.ddbj.nig.ac.jp/core/item/feature> ?CDS.
?CDS qualifier:location  ?location;
qualifier:gene ?gene;
qualifier:locus_tag      ?locus_tag;
qualifier:product        ?product;
qualifier:note   ?note;
qualifier:db_xref        ?db_xref.
}

#case7
prefix qualifier:<http://sabi.ddbj.nig.ac.jp/core/qualifier/>
select distinct  ?AccessNum
where{
?AccessNum ?p  ?feature.
?feature        qualifier:protein_id     'CAJ82835.1'.
}

#case8
prefix qualifier:<http://sabi.ddbj.nig.ac.jp/core/qualifier/>
select ?AccessNum ?features
where{
?AccessNum ?p  ?clone.
?AccessNum  ?has  ?features.
?clone  qualifier:clone  'TNeu120m03'.
}

#case9
prefix qualifier:<http://sabi.ddbj.nig.ac.jp/core/qualifier/>
select ?location ?gene ?locus_tag
where{
?CDS qualifier:location  ?location;
qualifier:gene ?gene;
qualifier:locus_tag      ?locus_tag;
qualifier:product        'adenosine kinase'.
}

#case10
prefix qualifier:<http://sabi.ddbj.nig.ac.jp/core/qualifier/>
select  ?lib
where{
?subject qualifier:clone         'TNeu120m03';
qualifier:clone_lib ?lib.
}
```
