# Supplementary material for: BioBenchmark Toyama 2012: an evaluation of the performance of triple stores on biological data
Source: J Biomed Semantics. 2014 Jul 10;5:32. doi: 10.1186/2041-1480-5-32 (PMC4118313; doi:10.1186/2041-1480-5-32)
Supplement: Additional file 2 — Configuration. This file presents the modified parameters for each database. [file 2041-1480-5-32-S2.pdf]

4store:

```
System:
vm.swappiness = 10
```

Bigdata:

```
com.bigdata.btree.writeRetentionQueue.capacity=50000
com.bigdata.rdf.sail.BigdataSail.bufferCapacity=1000000

System:
vm.swappiness = 10
```

Mulgara:

```
System:
vm.swappiness = 10
```

OWLIM-SE:

```
-Xmx60G -Xms30G -Druleset=empty -Dentity-index-size=800000000 -Dcache-memory=20000m
-Dtuple-index-memory=20000m -DenablePredicateList=false -Dpredicate-memory=0
-Dfts-memory=0 -DftsIndexPolicy=never  -Denable-context-index=false -Din-memory-literal-properties=false
-Dtransaction-mode=fast -Dtransaction-isolation=false -Drepository-type=file-repository

System:
vm.swappiness = 10
```

Virtuoso:

```
.ini file:
NumberOfBuffers          = 6500000
MaxDirtyBuffers          = 5000000
MaxCheckpointRemap       = 1000000
AsyncQueryMaxThreads     = 18
ThreadsPerQuery          = 18
IndexTreeMaps            = 512
ThreadCleanupInterval    = 1
ResourcesCleanupInterval = 1
Striping = 1
Segment1 = 10G, /data2/virtuoso/db-seg1-1.db, /data3/virtuoso/db-seg1-2.db
[HTTPServer]
ServerThreads               = 20
[Parameters]
ServerThreads               = 100

System:
vm.swappiness = 10
```
